# Educational inequalities in gastric cancer incidence and mortality, 1996–2015: a population-based study in Quito-Ecuador

**DOI:** 10.3332/ecancer.2026.2061

**Published:** 2026-01-19

**Authors:** Wilmer Tarupi, Esther de Vries, Patricia Cueva, Carol Guarnizo-Herreño

**Affiliations:** 1 Sociedad de Lucha contra el Cáncer, Quito Cancer Registry, Av Eloy Alfaro 5394 y los Pinos, Quito 170138, Ecuador; 2 Department of Clinical Epidemiology and Biostatistics, Pontificia Universidad Javeriana, Kra 7° #40-62, Bogotá, Colombia; 3 Sociedad Ecuatoriana de Oncología, Sociedad Ecuatoriana de Oncología, Quito 170521, Ecuador; 4 Departamento de Salud Colectiva, Facultad de Odontología, Universidad Nacional de Colombia, Carrera 45 # 26-85. Edif. Uriel Gutiérrez, Bogotá D.C., Colombia; a https://orcid.org/0000-0002-3611-7759; b https://orcid.org/0000-0002-5560-2258; c https://orcid.org/0000-0002-9320-3696; d https://orcid.org/0000-0002-8781-2671

**Keywords:** inequalities, socioeconomic, stomach, neoplasms, cancer, incidence, mortality, Ecuador

## Abstract

**Objective:**

To assess trends in educational inequalities in gastric cancer (GC) incidence and mortality in Quito, Ecuador, from 1996 to 2015.

**Methods:**

Using data available from the population-based cancer registry of Quito, age-specific standardised incidence and mortality rates for GC were calculated by educational level and sex. Using robust Poisson regression models, rate ratios, relative index of inequality (RII) and the slope index of inequality (SII) in incidence and mortality by educational level were estimated. Joinpoint regression analysis was performed to estimate the average annual percentage change.

**Results:**

The risk of developing GC among the less educated was substantially greater than among the most educated in both men and women, being around double the risk in relative terms (RII_women_ =2.1; 95% CI: 1.9, 2.3; RII_men_ = 1.7; 95% CI: 1.5, 1.8) and representing 14–15 cases per 100,000 person-years more cases in the lowest versus the highest educational levels (SII_women_ = 14.2; 95% CI: 12.2, 16.2; SII_men_ = 14.5; 95% CI: 12.0, 17.1). The differences were slightly greater for mortality.

**Discussion:**

Strong educational inequalities in GC incidence and mortality rates in Quito were observed. Efforts to target specific strategies among individuals with low education may help to ameliorate socioeconomic disparities in cancer incidence and mortality.

## Introduction

Gastric cancer (GC) remains the third most frequently diagnosed cancer and the leading cause of cancer-related deaths in Ecuador, with an incidence rate of 15.9 cases per 100,000 men and 9.6 cases per 100,000 women. In terms of mortality, the risk of dying from this disease is 12.9 cases per 100,000 men and 7.4 cases per 100,000 women, positioning Ecuador at a high level compared to other countries in the region [1]. Although incidence and mortality rates have declined since 1985, they have slowed down and stabilised since 2000. This stagnation, which has also been observed in other countries [2], alongside with a 5-year survival rate of 19% in recent years [3], makes it a public health priority, as it requires a specific institutionalised response.

In recent decades, research has shown that an individual's socioeconomic status significantly determines their risk of developing and dying from GC [4, 5]. Although most of the existing literature on this topic comes from Europe and the United States, where healthcare systems differ considerably from Ecuador's, it clearly demonstrates that individuals with lower socioeconomic status experience higher rates of illness, suffering and death from GC compared to those in higher socioeconomic positions [5]. The Ecuadorian healthcare system has a segmented and fragmented structure, where access to care varies based on an individual's ability to pay, potentially influencing the early detection of this disease.

Although the quantification, understanding and reduction of health inequalities have been recognised as strategic priorities in public policy formulation, a matter of social justice and human rights, as well as beneficial from an economic perspective [6, 8], data available on social inequalities in GC is scarce in Ecuador [5]. To understand inequalities in GC at a local level to better inform promotion and prevention efforts, we analysed trends in educational inequalities in GC incidence and mortality rates in Quito, from 1996 to 2015.

## Materials and methods

### Case definition

Data on GC cases and deaths (C16.0-9) from 1996 to 2015 were obtained from the Quito population-based cancer registry database, which records all diagnosed cancer cases and deaths reported in the urban area of the city. The registry, which only covers the urban area of the city, follows a methodology consistent with international standards set by the International Association of Cancer Registries as part of the International Agency for Research on Cancer [9–11]. During the study period, the coding system for oncological diseases (ICO-3) was updated from version 2 to version 3 in 2002. This transition allowed for a more precise classification and tracking of GC cases, including distinctions based on tumour location and histology. Data reporting adheres to the International Classification of Diseases, 10th edition. Gastric lymphomas were excluded from the analysis.

Data on age and sex were available for more than 99.0% of cases, while data on educational level were absent for 7.1% of the incident cases of cancer (Table 1). We analysed data of patients diagnosed in the following four periods: 1999–2000, 2001–2005, 2006–2010 and 2011–2015. During the first study period 1996–2000, the information on level of education was unavailable for 10% of the cases, which decreased progressively to 6% for the last period 2011–2015. Compared with the analytic sample, those excluded from the analysis because of missing data tended to be older (mean 69 versus 66 years; *p* < 0.01). No other significant difference was observed between those included and excluded from the analysis.

### Population

Data on mid-year population counts by age and sex were obtained from the 1990, 2001 and 2010 National Censuses [12-14]. For the remaining years, intercensal estimates were calculated using the geometric growth rate, which assumes constant compound growth over time. To estimate the annual population size for each educational group, the proportions of individuals at each education level, as derived from the census, were applied to the total projected population for each year, stratified by age and sex.

### Education level criteria

The educational level was defined as the highest level in which the individual enrolled during his/her life (i.e., the person accessed but not necessarily graduated at this level) and was classified into three groups: (a) primary education or less, (b) secondary education and (c) tertiary education (post-secondary instruction). Data on educational level were extracted from the cancer registry database, which contains this information from the medical records of each patient diagnosed with GC and subsequently verifies and validates it using information from the civil registry.

Socio-economic position (SEP) was operationalised using educational attainment. The education variable allows capturing the resources related to a person's knowledge and constitutes a good proxy of the SEP for life, since it is usually achieved relatively early in life. As such, education can capture both the circumstances of the SEP of family origin and personal, as it usually has a significant effect on the occupation and income [15, 16].

### Data analysis

Age-standardised incidence (ASIR) and mortality rates (ASMR) according to educational level were calculated using the direct method of standardisation, with Segi’s world standard population [17]. Rates are expressed per 100,000 person-years. The annual trends by sex and educational level were quantified by calculating the average annual percentage change (AAPC), using the Joinpoint Regression Program version 4.7.0.0 of the Surveillance Research Program of the National Cancer Institute of the United States [18]. The AAPC method uses the underlying Joinpoint model to compute a summary measure over a fixed, pre-specified interval.

We implemented robust Poisson regression models stratified by sex with the number of cases of incidence and mortality as a dependent variable and the natural logarithm of person-years as the offset, incorporating educational level as an independent variable. First, we estimated the incidence and mortality rate ratios (RR) by educational level, which compared the rates of the primary and secondary educational groups with the rate in the tertiary education group (reference group). Changes in RR over time are the result of changes in the risks and the distribution of the educational levels. To assess changes in the magnitude of inequalities, in both relative and absolute terms, controlling for changes in the educational distribution over time, we estimated the relative index of inequality (RII) and the slope index of inequality (SII), which were derived after transforming the education variable into an educational rank, considering the size of each educational group [19, 20].

## Results

A total of 4,638 cases of GC were diagnosed between 1996 and 2015 (2,491 men, 2,147 women). Over time, the general trend of ASIR showed a slight, non-significant increase in both men and women. ASIR changed from 23.7 to 24.6 cases in men and from 13.0 to 17.1 per 100,000 population in women, but these increases were not statistically significant (AAPC_men_ = 0.35; 95% CI: −0.4, 1.1; AAPC_women_ = 1.0; 95% CI: −0.1, 2.2). Men had overall higher incidence rates than women throughout the study period.

**Figure 1.**
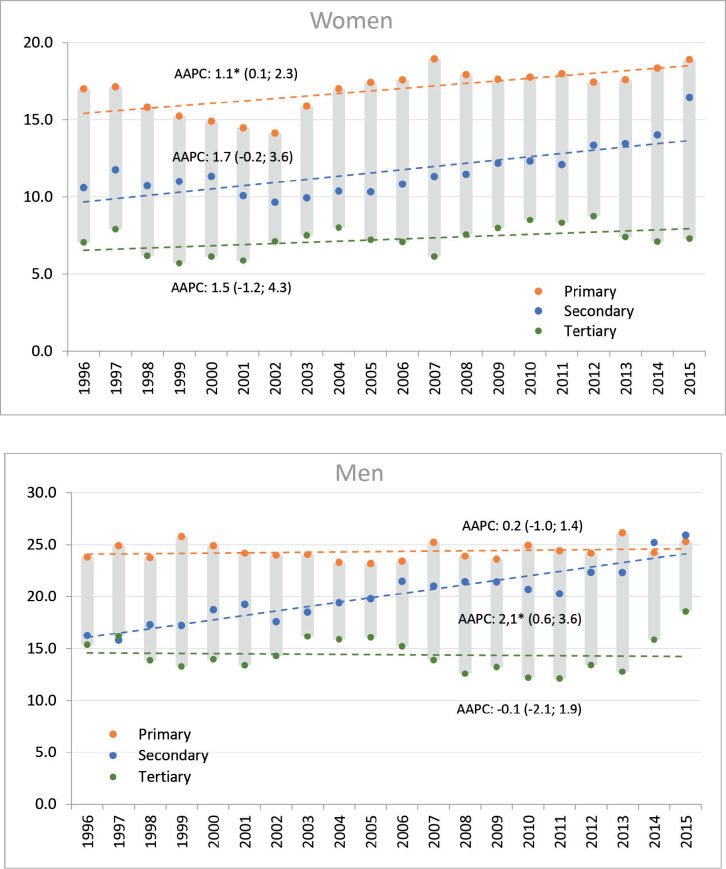
Trends in standardised incidence rates of gastric cancer, including the AAPC based on the joinpoint model by sex and educational level. Legend: Y: Age-standardised rates per 100,000 person-years, X: Year of diagnosis; AAPC: Average Annual Percent Change, figures between brackets are 95% CI of AAPC; a star indicates statistical significance at *α* = 0.05. Colours indicate groups of educational level.

The general trend towards increasing incidence rates was not reflected equally across levels of education. Among men, a non-significant decrease was observed in the higher educational level, a significant and sustained increase at the secondary level and a non-significant increase at the primary educational level. While in women, the increase in incidence rates was observed in all educational levels, being significant only in the lower level of education (Figure 1).

**Figure 2.**
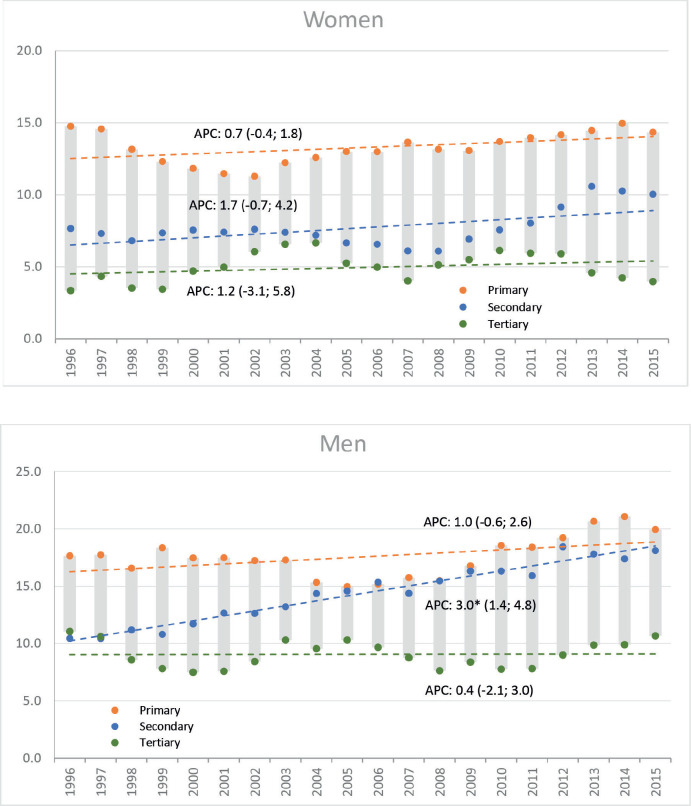
Trends in standardised mortality rates gastric cancer, including AAPC based on the joinpoint model by sex and educational level. Legend: Y: Age-standardised rates per 100,000 person-years; X: Year of death; AAPC: Average Annual Percent Change, figures between brackets are 95% CI of AAPC; a star indicates statistical significance at *α* = 0.05. Colours indicate groups of educational level.

During the same period, a total of 3,634 deaths were registered (1,947 men, 1,687 women). ASMR increased significantly from 13.4 to 17.6 deaths in men (AAPC: 1.1; 95% CI: 0.1, 2.2) and non-significantly in women; from 11.1 to 11.9 deaths per 100,000 (AAPC: 0.5; 95% CI: −0.5, 1.5). Like the incidence, the increasing trend in mortality among men was only observed in the middle-educated group. In women, stable rates were observed in all educational levels (Figure 2).

During the whole study period, RRs showed a higher risk of developing GC among the less educated compared to the more educated adults, taking the pattern of a social gradient. This association was stronger in women (RR_primary_ 6.4, RR_secondary_ 3.0, both *p* < 0.0001) than in men (RR_primary_ 3.1, RR_secondary_ 1.5, both *p* < 0.0001). A similar picture was observed for mortality rates, women (RR_primary_ 7.6, RR_secondary_ 3.1, both *p* < 0.0001) and men (RR_primary_ 4.0, RR_secondary_ 1.8, both *p* < 0.0001). The RII for the incidence of GC in men was 1.7 (95% CI: 1.5, 1.8) and 2.1 (95% CI: 1.9, 2.3) in women (Table 2). For GC mortality, the RII was 2.1 (95% CI: 1.8, 2.2) in men and 2.4 (95% CI: 2.3, 2.6) in women (Table 3).

In absolute terms, for the entire study period, it was evidenced that women at the lowest educational level experienced 14.2 (95% CI: 12.2; 16.2) more cases per 100,000 than women with higher educational levels. While, for men, at the lower educational level, there would be 14.5

(95% CI: 12.0; 17.1) more cases per 100,000 than at the higher educational level (Table 2). Regarding mortality, for the entire study period, women at the lowest educational level had 12.6 deaths per 100,000 more than women with higher educational levels. For men, those with lower educational levels had 12.4 more deaths per 100,000 than men with higher educational levels (Table 3).

## Discussion

We observed strong educational inequalities in the incidence and mortality rates for GC in Quito, which were persisted throughout the study period (1996–2015), with a social gradient of increasing risk of developing and dying from GC as the educational level decreases. This pattern suggests the presence of persistent structural inequities that transcend individual-level factors. Whereas GC incidence and mortality were higher among men, educational inequalities tended to be stronger among women. The results are in line, also in terms of magnitude and direction, with those reported in several studies, in which the risk of developing and dying from GC was related to populations with low educational levels [4, 5, 21–25].

Considering that the GC is preceded by a prolonged precancerous process that lasts around two decades [26], the observed inequalities in our study are likely related to actions that occurred in the last few decades: from individual and collective behaviours, customs and social interactions linked to the exposure to cancer risk factors, to the availability and access to early detection, timely diagnosis and effective treatments [27]. Such long latency offers an important window to intervene, but also reflects the cumulative effects of social disadvantage over time. Notably, during the study period and even before, Ecuador lacked established national strategies for the prevention and control of GC within its laws and public health policies [28], which may have disproportionately affected lower socioeconomic groups.

Women with the lowest educational level had a 2.1-fold higher risk of developing GC than women with higher education, while men had a 1.7-fold higher risk. These findings are consistent with those from a meta-analysis, which found that individuals with lower educational levels had a significantly higher risk of developing GC (RII = 2.97) [24]. It also coincides with studies in Colombia, where the RII for women was 3.2 and for men 2.1 between 1998 and 2007 [21]. On the other hand, while the mortality rate among men and women with high educational levels was 9.1 and 4.9 cases per 100,000, respectively, those with lower educational levels faced nearly double the rates (17.5 and 13.2 per 100,000, respectively). This trend is also observed in countries such as India, Brazil, Mexico and Colombia [5, 25].

The findings suggest that more advantaged individuals may be relatively protected against GC, likely due to differences in exposure to cancer risk factors, such as smoking, alcohol, obesity, *Helicobacter pylori* infection, occupational risk factors, housing circumstances and healthcare utilisation [29]. These factors are not randomly distributed, but deeply embedded in social structures, reinforcing the relevance of structural determinants. From a public health perspective, addressing the structural social determinants of GC is critical, as these shape the conditions in which people produce and reproduce their lives. Education in particular, implies the availability of knowledge resources that can be utilised to maximise health [30]. These resources, in turn, influence a variety of capacities such as financial means [31]; stable employment [31]; health literacy [32]; psychosocial factors such as self-esteem and sense of coherence [32]; being receptive to prevention messages [33]; being able to change health behaviours [33]; and making proper use of health care services [32, 33]. Moreover, low-educated people are more likely to have lower levels of social support and to have less control over their lives [34].

Regarding gender differences, men consistently had higher incidence rates than women throughout the study period, with a sex ratio that went from 1.5:1 to 1.4:1. This pattern is commonly observed globally, where men’s GC incidence rates are 1.4 to 2.3 times higher than those of women [35]. Some researchers have found statistical associations with smoking and suggest that this has contributed to a certain extent to the variation in gender rates [36]. Another element that determines the higher proportion of cases among men is people's attitude towards health care. When men and women face the same disease, the former tend to use health care services to a lesser extent [37], suggesting that health promotion and prevention strategies should be oriented from a gender perspective.

Ecuador's health system is fragmented and segmented into public, social security and private sectors, each managing its own financing, service delivery and resource allocation. In terms of cancer care, the private sector's capacity exceeds that of both the public sector and social security [38], a disparity that affects care pathways and exacerbates health inequities. Additionally, there are imbalances in the allocation of resources across these sectors, leading to minimal offerings for the poorest populations, which fail to meet patients' needs [39]. The issue lies not only in the limitations of structural segmentation regarding the coverage of various population groups, but also in the fragmentation of benefits received, due to the differing financial models of public and private entities [40]. A 2013 evaluation of oncological care in Latin America highlighted the negative impact of such a segmented and fragmented structure on access to diagnosis and treatment. It also demonstrated the consequences on the right to health, as delays in diagnosis and treatment initiation—common in such systems—are linked to advanced-stage cancer and higher mortality rates [41].

According to information published by the Quito Cancer Registry [42], 60.5% of cases were diagnosed at stage IV between 2003 and 2005, 58.0% between 2006 and 2010 [43] and 58.5% between 2011 and 2015 [44]. This trend has persisted in the years following the study period; between 2015 and 2019, the proportion of cases diagnosed at an advanced stage was 60.5% [45], when therapeutic options are limited. The persistent high proportion of late-stage diagnoses suggests a systemic failure in early detection, which is likely influenced by socioeconomic and educational inequalities. The behaviour of stage in relation to social position, as well as cancer survival, is something that requires further exploration.

Neither before nor during the study period were there organised national screening programs [28, 46]. The public health system offered opportunistic or spontaneous screening to subjects who voluntarily go to health services, either for the purpose of undergoing a screening test or because of symptoms [46]. Our findings suggest that individuals with higher levels of education were relatively protected from GC, as they may have higher cancer health literacy necessary for making informed decisions regarding cancer prevention, diagnosis and treatment. Cancer health literacy has been associated with educational level and determining in the clinical setting the doctor-patient relationship and impacting oncology care. This association underscores the role of education not only as a social determinant but also as a functional enabler of health-related decision-making [47].

It was only in 2017 that a national standard was published. The National Strategy for Comprehensive Cancer Care recommends screening for individuals over 50 years of age using a combination of serological tests to detect *Helicobacter pylori* and pepsinogen levels, along with upper gastrointestinal endoscopy. The frequency of endoscopic studies is determined using the ABC stratification method [46]. While these measures could help control GC, it is crucial to reassess the strategies considering social inequities, focusing on early detection and timely diagnosis, particularly for disadvantaged populations, to enhance cancer control efforts. Given the high incidence rates, widespread implementation of *H. pylori* screen-and-treat strategies may be more cost-effective if the intervention targets high-risk groups. However, successful implementation requires adequate resources, effective treatments and proper infrastructure. Conducting a pilot study and cost-effectiveness analysis is crucial to assess the program's feasibility and prioritise resources [48]. Such approaches must explicitly incorporate equity as a guiding principle to avoid further widening health gaps.

The strength of this research lies in its use of Ecuador's oldest population-based cancer registry [49], recognised for its high-quality data and international standards [10]. The inclusion of education level data in the registry is a significant advantage, as education is a stable indicator with a strong correlation to other socio-economic factors such as occupational status and income [16]. Moreover, education is less susceptible to reverse causality compared to other SEP indicators, as it is typically established early in life [50]. However, a limitation is the potential bias introduced by excluding participants with missing education data. This caused those included in the analysis being slightly younger than those excluded, which may have led to an underestimation of rates and a bias in the results, particularly those related to the association between education and GC incidence and mortality. Nevertheless, the consistency of findings and their alignment with international literature strengthen the internal validity and external plausibility of our results.

To the best of our knowledge, this is the first published study in Latin America to investigate long-term trends in GC incidence and mortality by education level using data from a population-based cancer registry, offering a unique opportunity to analyse educational disparities in GC through registry data. This analysis provides essential evidence to guide future interventions aimed at reducing social inequalities in cancer outcomes.

## Conclusion

We found that GC incidence and mortality were inversely associated with educational attainment. Efforts to target specific strategies among individuals with low education may help to ameliorate socioeconomic inequalities in cancer incidence and mortality, but must be accompanied by broader structural reforms to ensure sustainability and effectiveness. From a public policy perspective, a comprehensive national cancer control plan that includes actions on the social determinants, specific risk factors and early detection represents the most impactful and equitable approach to reducing these inequalities over time.

## Conflicts of interest

The authors declare they have no known competing financial interests or personal relationships that could have appeared to influence the work reported in this paper.

## Funding

This research did not receive any specific grant from funding agencies in the public, commercial or not-for-profit sectors.

## Ethics approval and consent to participate

This article is based on secondary analysis of anonymised data on cases/deaths and population counts in aggregate form. Data on population are publicly available by the National Institute of Statistics and Census, while data on cases and deaths, from the Population-Based Cancer Registry of Quito required an approval of Human Research Ethics Committee of Oncological Hospital SOLCA Quito. Reference number: CEISH SOLCA.OBS 17.014. Approval date: April 2018. All procedures followed were in accordance with the ethical standards of the responsible committee on human research and in compliance with the Helsinki Declaration of 1964 and later versions.

## Disclosure

This study was previously presented at the International Association of Cancer Registries (IACR) Annual Scientific Virtual Conference, November 2020, where it received the Sharon L. Whelan Special Prize, awarded by the International Agency for Research on Cancer (IARC). A summarised version was published as an abstract at the 40th IARC Annual Scientific Conference in Arequipa, Peru, 2018 [51].

## Author contributions

Wilmer Tarupi: Conceptualisation, Methodology, Data curation, Formal analysis, Writing – original draft, Visualisation, Writing – review & editing, Supervision.

Esther de Vries: Investigation, Methodology, Writing – review & editing, Supervision.

Patricia Cueva: Investigation, Methodology, Data curation, Writing – review & editing.

Carol Guarnizo-Herreño: Methodology, Writing – review & editing.

## Figures and Tables

**Table 1.** Missing values of the education variable, by period and sex.

Sex	Period	Cases	Analytic sample	Missing values	
				*n*	%
Men	1996–2000	513	469	44	8.6
	2001–2005	684	639	45	6.6
	2006–2010	691	640	51	7.4
	2011–2015	787	743	44	5.6
Women	1996–2000	461	408	53	11.5
	2001–2005	539	500	39	7.2
	2006–2010	596	560	36	6
	2011–2015	722	679	43	6
Total		4,993	4,638	355	7.1

**Table 2.** Standardised incidence rate (ASIR), rate ratio (RR) and relative index of inequality (RII) and slope index of inequality (SII) of gastric cancer in the adult population of Quito, by sex and level of education, 1996–2015.

Sex	Period	Education	Cases	ASIR	RR (95% CI)	RII (95% CI)	SII (95% CI
Men	1996–2000	Primary	279	23.7	3.6 (2.8; 4.6)	1.5 (1.1; 1.8)	14.8 (1.7; 15.2)
		Secondary	113	17.3	1.4 (1.1; 1.9)		
		Tertiary	77	13.9	1		
	2001–2005	Primary	369	24.0	3.3 (2.7; 4.1)	1.4 (1.0; 1.7)	11.7 (10.5; 13.0)
		Secondary	159	18.5	1.4 (1.1; 1.8)		
		Tertiary	111	16.2	1		
	2006–2010	Primary	336	23.9	2.9 ( 2.4; 3.6)	2.3 ( 2.0; 2.6)	16.5 (9.5; 23.6)
		Secondary	190	21.4	1.7 (1.3; 2.1)		
		Tertiary	114	12.6	1		
	2011–2015	Primary	378	25.4	2.8 (2.3; 3.4)	1.7 (1.4; 1.9)	14.4 (7.4; 21.5)
		Secondary	230	23.6	1.7 (1.3; 2.1)		
		Tertiary	135	15.2	1		
	1996–2015	Primary	1,362	24.3	3.1 ( 2.8; 3.5)	1.7 (1.5; 1.8)	14.5 (12.0; 17.1)
		Secondary	692	20.2	1.5 (1.4; 1.7)		
		Tertiary	437	14.5	1		
Women	1996–2000	Primary	261	15.8	7.9 (5.5; 11.3)	1.9 (1.5; 2.3)	13.2 (11.2; 15.3)
		Secondary	114	10.7	3.4 (2.3; 5.0)		
		Tertiary	33	6.2	1		
	2001–2005	Primary	330	15.9	7.6 (5.6; 10.5)	2.1 (1.7; 2.4)	12.4 (11.5; 13.1)
		Secondary	127	9.9	2.9 (2.1; 4.1)		
		Tertiary	43	7.5	1		
	2006–2010	Primary	348	17.9	6.5 (4.9; 8.7)	2.6 (2.3; 2.9)	15.6 (14.9; 16.3)
		Secondary	159	11.5	3.0 (2.1; 4.0)		
		Tertiary	53	7.6	1		
	2011–2015	Primary	380	18.3	4.9 (3.8; 6.2)	2.1 (1.8; 2.3)	14.8 (10.1; 19.5)
		Secondary	221	14.5	2.8 (2.1; 3.6)		
		Tertiary	78	7.7	1		
	1996–2015	Primary	1,319	17.0	6.4 (5.5; 7.3)	2.1 (1.9; 2.3)	14.2 (12.2; 16.2)
		Secondary	621	11.7	3.0 (2.5; 3.5)		
		Tertiary	207	7.2	1		

Legend: Primary = up to elementary or primary school; secondary = any level of high school; tertiary = any level of postsecondary education after high school including college and university. All estimates were calculated using robust Poisson regression models

**Table 3.** Standardised mortality rate (ASMR), rate ratio (RR), relative index of inequality (RII) and slope index of inequality (SII) of gastric cancer in the adult population of Quito, by sex and level of education, 1996–2015.

Sex	Period	Education	Deaths	ASMR	RR (95% CI)	RII (95% CI)	SII (95% CI)
Men	1996–2000	Primary	193	16.6	4.8 (3.4; 6.7)	1.8 (1.3; 2.2)	11.9 (11.5; 12.3)
		Secondary	75	11.2	1.8 (1.3; 2.7)		
		Tertiary	40	8.6	1		
	2001–2005	Primary	305	17.3	4.4 (3.4; 5.7)	1.8 (1.4; 2.1)	10.2 (9.6; 10.9)
		Secondary	113	13.2	1.6 (1.2; 2.2)		
		Tertiary	69	10.3	1		
	2006–2010	Primary	309	15.5	4.0 (3.1; 5.2)	2.7 (2.4; 3.0)	10.9 (2.7; 19.1)
		Secondary	149	15.5	1.9 (1.5; 2.5)		
		Tertiary	76	7.6	1		
	2011–2015	Primary	331	20.7	3.3 (2.6; 4.2)	1.9 (1.6; 2.3)	15.4 (9.3; 21.5)
		Secondary	189	17.8	1.9 (1.5; 2.4)		
		Tertiary	98	9.9	1		
	1996–2015	Primary	1,138	17.5	4.0 (3.5; 4.5)	2.1 (1.8; 2.2)	12.4 (8.6; 16.3)
		Secondary	526	14.4	1.8 (1.6; 2.1)		
		Tertiary	283	9.1	1		
Women	1996–2000	Primary	211	13.2	10.5 (6.7; 16.6)	2.3 (1.8; 2.7)	14.1 (13.7; 14.6)
		Secondary	75	6.8	3.7 (2.3; 6.1)		
		Tertiary	20	3.5	1		
	2001–2005	Primary	248	12.2	7.0 (4.9; 10.1)	2.1 (1.7; 2.5)	8.3 (6.1; 10.5)
		Secondary	91	7.4	2.6 (1.7; 3.8)		
		Tertiary	35	6.6	1		
	2006–2010	Primary	311	13.2	7.9 (5.7; 11.1)	3.0 (2.6; 3.4)	13.6 (10.9; 16.2)
		Secondary	110	6.1	2.8 (1.9; 4.0)		
		Tertiary	39	5.1	1		
	2011–2015	Primary	329	14.4	6.7 (4.9; 9.0)	2.4 (2.0; 2.7)	13.7 (9.5; 18.0)
		Secondary	169	10.6	3.4 (2.5; 4.7)		
		Tertiary	49	4.6	1		
	1996–2015	Primary	1,099	13.2	7.6 (6.4; 9.1)	2.4 (2.3; 2.6)	12.6 (10.6; 14.6)
		Secondary	445	7.7	3.1 (2.5; 3.7)		
		Tertiary	143	4.9	1		

Legend: Primary = up to elementary or primary school; secondary = any level of high school; tertiary = any level of postsecondary education after high school including college and university. All estimates were calculated using robust Poisson regression models
